# Supplementary material for: Interplay of Structural Factors in Formation of Microphase-Separated or Microphase-Mixed Structures of Polyurethanes Revealed by Solid-State NMR and Dielectric Spectroscopy
Source: Polymers (Basel). 2021 Jun 14;13(12):1967. doi: 10.3390/polym13121967 (PMC8232235; doi:10.3390/polym13121967)
Supplement: Supplementary file 1 [file polymers-13-01967-s001.zip › polymers-1245269-supplementary.pdf]

Supplementary Materials

1. Dielectric data processing

Complex dielectric permittivity $\varepsilon^*(f) = \varepsilon'(f) - i\varepsilon''(f)$ was measured isothermally in steps of 5°C in temperature interval -150 to + 200°C and in the frequency range from 10^{-2} to 10^6 Hz. The complex dielectric modulus M* is related to ε* as follows [1]:

$$M^* = \frac{1}{\varepsilon^*} = M' + iM'' \quad (1)$$

$$M' = \frac{\varepsilon'}{\varepsilon'^2+\varepsilon''^2} \text{ and } M'' = \frac{\varepsilon''}{\varepsilon'^2+\varepsilon''^2} \quad (2)$$

We chose to apply the straightforward derivative method where the ohmic-conduction-free loss is determined from the logarithmic derivative of the dielectric constant [2]:

$$\varepsilon''_D = -\frac{\pi}{2}\frac{\partial \varepsilon'\{\omega)}{\partial \ln \omega} \quad (3)$$

It was shown that this method is a very good approximation of the "conduction-free" loss for relatively broad loss peaks like those observed here, while narrow peaks appear much narrower in ε''_D than in ε'' [2]. In order to evaluate the individual relaxation processes quantitatively, a model function has been fitted to the dielectric data, with the Havriliak- Negami phenomenological relation [3].

Traditional Havriliak- Negami Equation:

$$\varepsilon*(\omega) = \varepsilon_\infty + \frac{\Delta\varepsilon}{(1+(i\omega\tau_{NH})^{1-\alpha})^\beta} \quad (4)$$

being the most general form. In this expression, $\varepsilon^* = \varepsilon' - i\varepsilon''$, is the complex dielectric function, $\omega = 2\pi f$, f is the field frequency, $\Delta\varepsilon$ is the intensity of the dielectric process, $\tau_{NH} = 1/2\pi f_{NH}$ and f_{NH} is the position of the relaxation process on the frequency scale, ε_∞ is ε'(f) for $f >> f_{NH}$, α and β are shape parameters representing the symmetrical and asymmetrical broadening of the relaxation with respect to the Debye peak. The main characteristic of each relaxation process is the most probable relaxation time, τ_{max}, determined according to [4] as

$$\tau_{max} = \tau_{HN}\left(\frac{\sin\left(\pi\alpha\beta/_{2(\beta+1)}\right)}{\sin\left(\pi\alpha/_{2(\beta+1)}\right)}\right)^{1/\alpha} \quad (5)$$

The temperature dependence of lokal relaxations was modeled by an Arrhenius type expression (Eq.6) [5]:

$$\tau(T)_{max} = \tau_0 \exp\left(E_a/_{RT}\right) \quad (6)$$

Here $\tau_0 = \tau_{max}$ at $T \rightarrow \infty$, E_a is the activation energy.

The α-relaxation is connected with onset of large-scale motions of the chain segments in the vicinity of T_g. The temperature dependence of the characteristic relaxation times have been described using the Vogel-Fulcher-Tammann equation [5] (Fig.10):

$$\tau = \tau_0 \exp\left(\frac{DT_0}{T-T_0}\right) \quad (7)$$

Where τ_0 is the relaxation time at infinite high temperature, T_0 is so-called Vogel temperature at which the relaxation time goes to infinity, and D is the parameter related to the *fragility* of material [6]. A smaller value of D implies steeper temperature dependence of the relaxation time or a more “fragile” behavior. The D data are given in Table 2. According to Plazek et al. [7,8] the activation energy of α- relaxation process was calculated using the following equation:

$$\frac{E_a}{R} = \frac{DT_0}{(1-\frac{T_0}{T_g})^2} \quad (8)$$

where E_a is the activation energy, R is the gas constant and T_g the glass transition temperature. The values of D and T_o parameters were extracted from the best fit to equation (5). The values of activation energies for α- relaxation as well as the VFT parameters T_o, D and T_g are gathered in Table 3.

References

(1) H.W. Starkweather Jr, P. Avakian, Conductivity and the electric modulus in polymers. J. Polym. Sci. Part B Polym. Phys. 30 (1992) 637-641.

(2) A.M. Castagna, D. Fragiadakis, H.K. Lee, T. Choi, J. Runt, The role of hard segment content on the molecular dynamics of poly(tetramethylene oxide) –based polyurethane copolymers. Macromolecules, 44 (2011) 7831-7836.

(3) S. Havriliak, S. Negami, A complex plane analysis of α-dispersions in some polymer systems. J. Polym. Sci. Part C 14 (1966) 99-117.

(4) S. Havriliak, S. Negami, A complex plane representation of dielectric and mechanical relaxation processes in some polymers. Polymer 8 (1967) 161-210.

(5) F. Kremer, A. Schonhals, Broadband dielectric spectroscopy. Springer, New York, 2003.

(6) C.A. Angell, K.L. Ngai, G.B McKenna, P.F. McMillan, S.W. Martin, Relaxation in glassforming liquids and amorphous solids. J. Appl. Phys. (2000) 883113 doi:

10.1063/1.1286035.

(7) D.J. Plazek, J.H. Magil, Physical properties of aromatic hydrocarbons I. J. Chem. Phys. 35 (1998) 3038-3050.

(8) E, Bureau, C. Cabot, S. Marais, J.M. Saiter, Study of the α-relaxation of PVC, EVA and 50/50 EVA_{70}/PVC blend. Eur. Polym. J. 41 (2005) 1152- 1158.

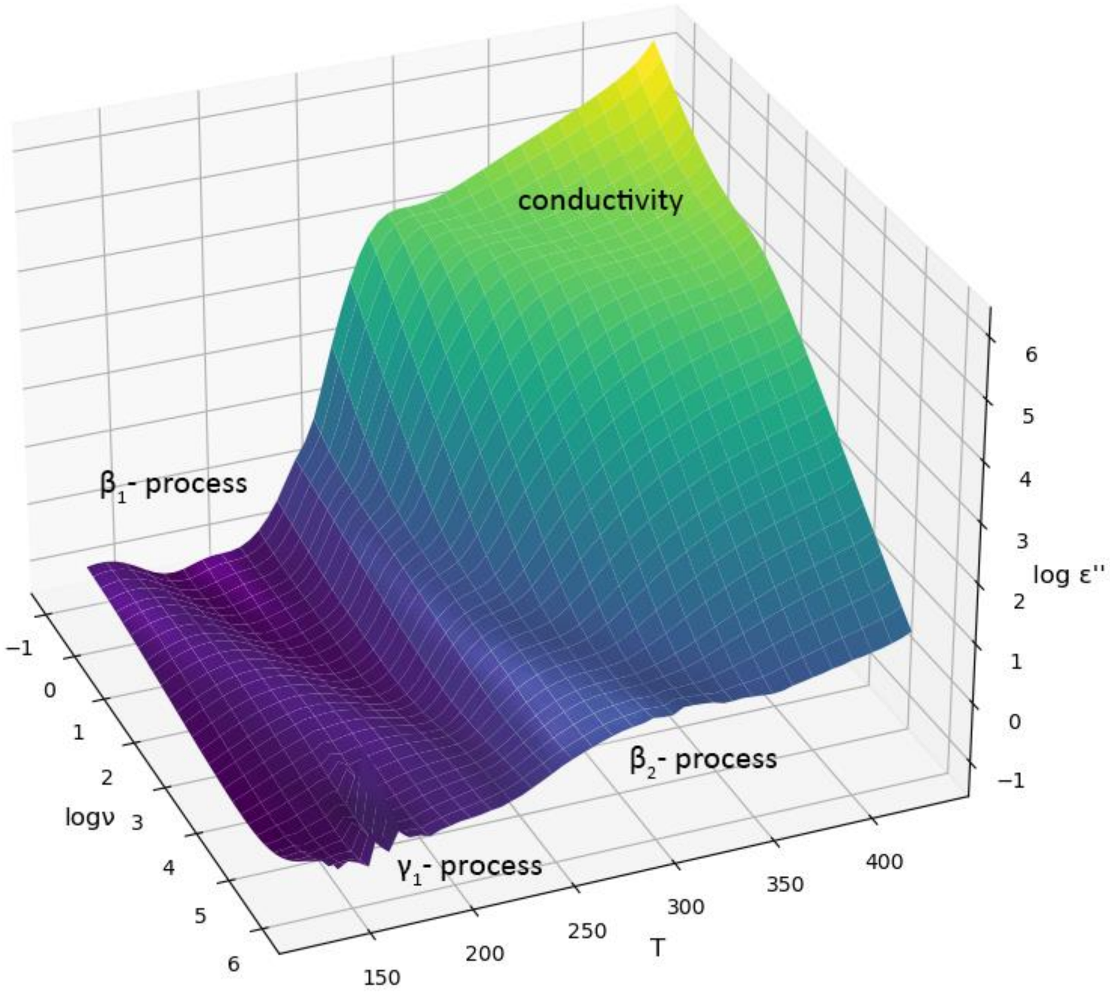

Figure S1. 3D plot of log ε” versus frequency and temperature for sample R_1/100.

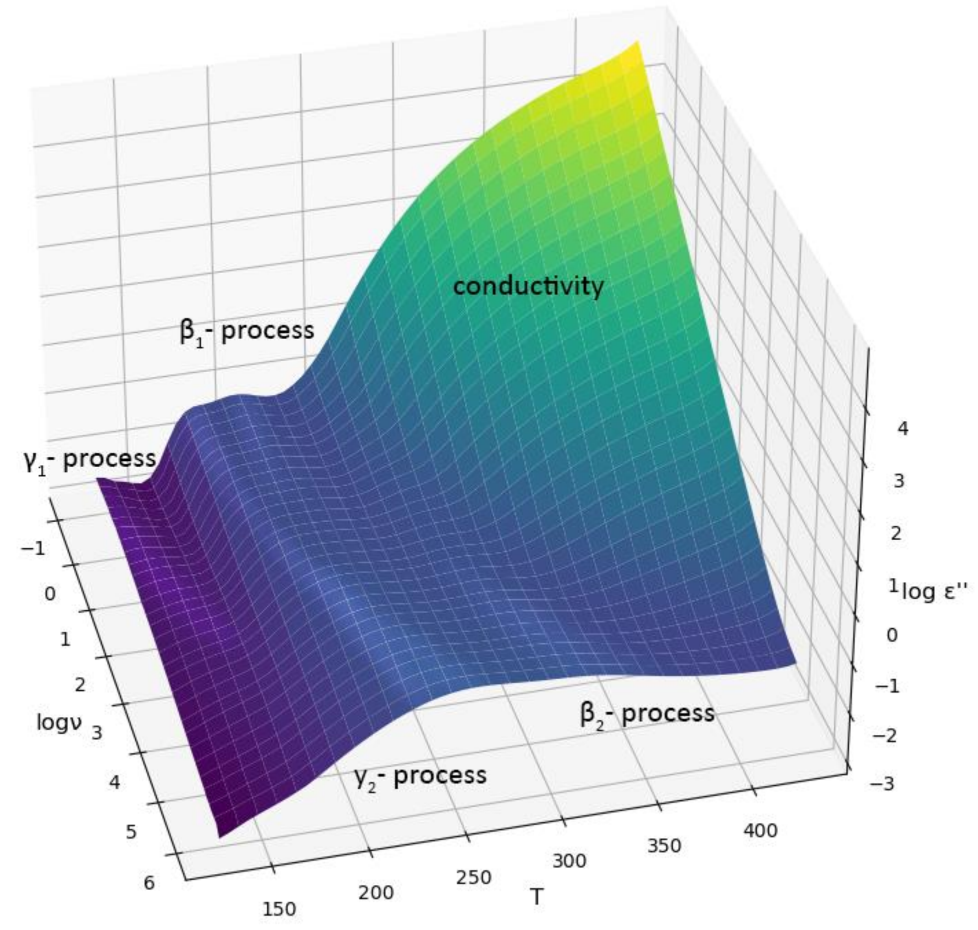

Figure S2. 3D plot of log ε” versus frequency and temperature for sample R_1/50/R_4/50.

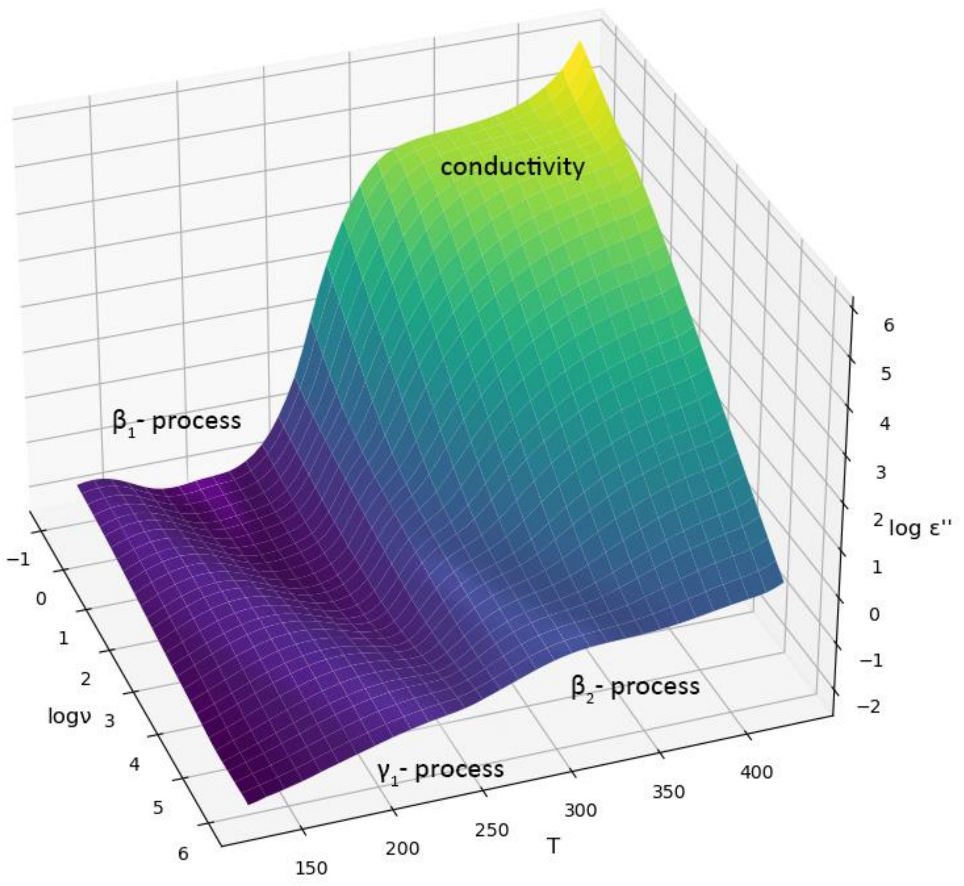

Figure S3. 3D plot of log ε'' versus frequency and temperature for sample R_1/50/R_3/50.

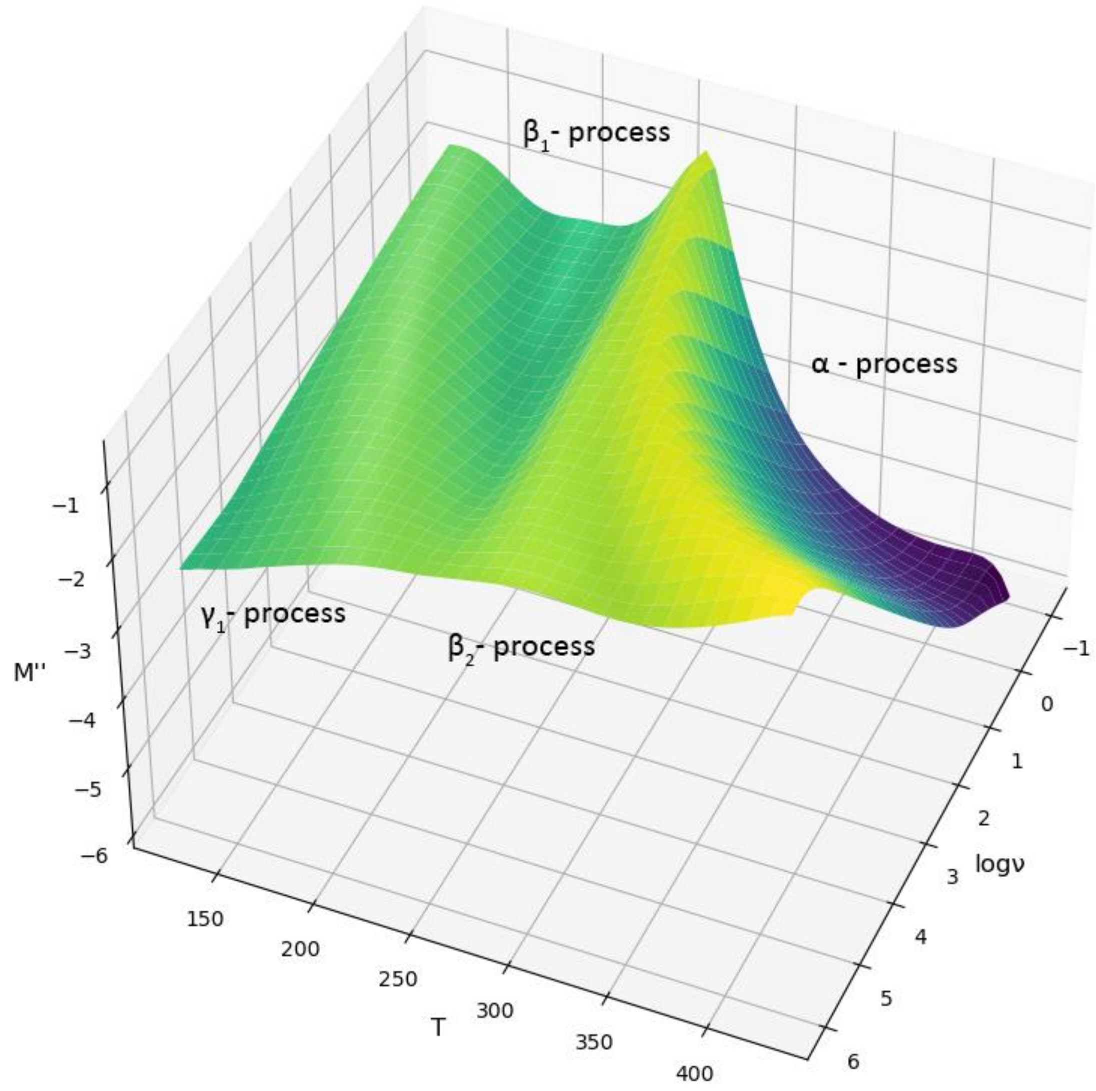

Figure S4. 3D plot of M" versus frequency and temperature for sample $R_1/50/R_3/50$.

NMR experiments

NMR experiments were performed on a Bruker Avance II 500 spectrometer operating at a proton frequency of 500.13 MHz. 4 mm and 7 mm double resonance MAS probe heads were used for recording ^{13}C spectra under MAS conditions and for ^{1}H static spin-diffusion experiments correspondingly.

(1) CP-MAS experiments. Conventional cross-polarization MAS NMR experiment (CP-MAS) with high power proton decoupling (100 kHz) was used to detect the signals from rigid component with high CP efficiency, recycle delay was 5 s, CP contact time - 100 μs. ^{13}C direct-polarization MAS NMR experiment with echo detection (90˚-τ-180˚-τ-aq) and low power proton decoupling

(LPD, 25 kHz) was used to observe the mobile component. Applying 180˚ pulse after τ delay refocuses mobile component whereas the broad signals from the rigid part are already irreversibly relaxed.[1] Recycle delay in this experiment was 0.5 s (optimal for short relaxing of mobile component), echo delay τ - 3ms. Spinning frequency in all experiments was 5-8 kHz. Spectra were recorded at ambient temperature.

(2) CPSP-MAS experiments. This method combines both CP and single pulse (SP) in one experiment, leading to a hybrid NMR spectrum[2]. Pulse sequence requires only one additional 90° pulse on ^{13}C channel before CP stage. As a result, such scheme overcomes a very long ^{13}C relaxation delay required by SP experiment for the rigid phases and low efficiency of CP to the mobile phases.

(3) ^{1}H DQ build-up and spin-diffusion experiments. The method used for ^{1}H DQ spin-diffusion measurements was the same as the one suggested by Saalwachter et al[3] which we used in our previous work. [4] The spectral width was 200 ppm (100 kHz) to cover the broad hard-phase (HS) signal. Pulse sequence is a conventional 5-pulse DQ experiment with a variable z-filter with proper phase cycling for DQ filtration.[5] DQ build-up curves were measured using the same scheme by varying excitation/reconversion time with additional 180° pulses in excitation and reconversion segments to compensate for spectral offsets and chemical-shift dispersion at long evolution times.[6] For spin-diffusion experiments, the DQ excitation and reconversion time τ_{DQ} of 15 μs was used to obtain maximum hard-phase signal with negligible inter- and mobile-phase magnetization, because it corresponds to the maxima of the DQ build-up curves at short times (the range of the strong ^{1}H dipolar interactions). For the separate integration of individual resolved mobile signals a narrow 20 ppm region around the sharp signals was baseline-corrected using a second order polynomial, thus removing the HS background hump. These integrations yield the overall mobile fraction (SS). All spin diffusion experiments in this work were performed under static conditions to avoid any influence of MAS on the spin diffusion coefficients.[7]

(4) ^{1}H Hahn echo NMR experiment was employed to measure the transverse magnetization relaxation (T_2 decay). Additional experiments with the dipolar filter before echo sequence were performed to obtain the T_2 of the mobile component directly,[7] which could be used to determine the proton spin diffusion coefficient for the mobile polymers as proposed by Mellinger et al.[8] The experimental conditions of the dipolar filter sequence before Hahn echo were the same as used in corresponding ^{1}H dipolar filter experiments.

(5) WISE experiments. Wideline separation (WISE) two-dimensional NMR experiment was used to characterize different dynamics in polymer systems – molecular motion is probed by ^{1}H wide-line shapes, which are separated in the second dimension by the ^{13}C chemical shift.[9] The pulse sequence for the dipolar WISE experiment is similar to the original. The contact time was 500 µs, spin-diffusion mixing time, tm, was 100 µs. A MAS spinning speed of 5 kHz and recycle delay 3 s were applied in all experiments.

(1) Shu, J.; Li, P.; Chen, Q.; Zhang, S. Quantitative measurement of polymer compositions by NMR spectroscopy: targeting polymers with marked difference in phase mobility, Macromolecules 2010, 43, 8993–8996.

(2) Mauri,M.; Thomann, M.Y.; Schneider,H.; Saalwachter,K. Spin-diffusion NMR at low field for study of multiphase solid, Solid State Nucl. Magn. Reson. 2008, 34, 125-141.

(3) Saalwachter,K. Proton multi-quantum NMR for the study of chain dynamics and structural constraints in polymeric soft materials, Prog. Nucl. Magn. Res. Spect. 2007,51, 1-35.

(4) Mokeev, M.V.; Ostanin, S.A.; Saprykina, N.N.; Zuev, V.V. Microphase structure of polyurethane-polyurea copolymers as revealed by solid-state NMR: effect of molecular architecture, Polymer 2018, 150, 72-83.

(5) Jia, Z.; Zhang, I.; Chen, Q.; Hansen, E.W. Proton spin diffusion in polyethylene as a function of magic-angle spinning rate. A phenomenological approach, J. Phys. Chem. A 2008,112, 1228–1233.

(6) Landfester, K; . Spiess, H.W. Characterization of interphases in core—shell latexes by Solid-State NMR, Acta Polymerica 1998, 49, 451–461.

(7) Nagapudi, K.: Leisen, J.; Beckham, H.W.; Gibson, H.W. Solid-state NMR investigations of Poly[(acrylonitrile)-rotaxa-(60-crown-20)], Macromolecules 1999, 32, 3025–3033.

(8) Mellinger, F.; Wilhelm, M.; Spiess, H.W. Calibration of 1H NMR spin diffusion coefficients for mobile polymers through transverse relaxation measurements, Macromolecules 1999, 32, 4686-4691.
(9) Schmidt-Rohr, K.; Spiess, H. W. Multidimensional Solid-State NMR and Polymers, Academic Press: New York, 1994.
(10) Duer, M.J. (Ed.), Solid-state NMR Spectroscopy. Principles and Applications, Wiley: New-York, Chapter 6, 2007.

Calculation of domain size

. Domain sizes are evaluated via spin diffusion NMR by measuring the rates of change of nuclear spin polarizations associated with the two phases in a PUs. When a step-function initial polarization gradient is imposed on a two-component system, the initial rate of exchange is directly proportional to the inter-component surface-area/volume ratio in the sample. From that ratio, the domain size can be extracted if the morphology is known (spheres, rods, lamellae). Proton spin-diffusion experiments with a DQ filter were used to achieve a magnetization modulation across a dynamically heterogeneous sample. The DQ filter selects the ^{1}H magnetization from rigid domains with lower molecular mobility (strong dipolar couplings). The following step is a “mixing” period (t_m), in which the remaining proton magnetization stored on the ±Z axis diffuses to neighboring spins and eventually reaches equilibration of the inhomogeneous magnetization distribution. The final step is the direct ^{1}H detection. Precision of ^{1}H spin diffusion NMR experiments as well as straightforward experimental protocols depends on specific properties of the systems under study. It can be tested by studying DQ buildup curves (Fig.3). The domain size of the discrete phase B in the two-phase A/B mixture d_{dis} can be determined by the following equation:

$$d_{dis}=\frac{(\rho_A^H\varphi_A+\rho_B^H\varphi_B)}{\varphi_A\varphi_B}\times\frac{4\varepsilon\varphi_B}{\sqrt{\pi}}\times\frac{\sqrt{D_AD_B}}{\rho_A^H\sqrt{D_A}+\rho_B^H\sqrt{D_B}}\times\sqrt{t_m^{s,0}} \qquad (1)$$

Here φ_m (m = A, B) are volume fractions of the mobile phase A and rigid phase B, ρ_A^H and ρ_B^H are proton densities, D_A and D_B are spin-diffusion coefficients of mobile and rigid phases. The ε represents dimensionality, we accept the value 2, because according to the all previous data PU have a cylindrical shape of phase domains The data used for calculation are given in the Table 3. To determine domain sizes we use initial rate approximation method - it has been shown that for short mixing times the signal is proportional to $(t_m^{s0})^{1/2}$ (the characteristic mixing time of spin-diffusion, see eq.2). This parameter can be determined from intersection of the linear extrapolation of the initial part of the spin diffusion curve and the equilibrium magnetization level occurs. Detailed description such approach can be found in [1,2]. $t_m^{s,0}$ is the characteristic mixing time of spin-diffusion introduced by Mellinger et al. [39], which can be determined from the intercept of extrapolated initial linear-slope with the X-axis in the spin-diffusion curves as shown in Fig. 5.

For the transformation of spin-diffusion data into reliable information about spatial structure of PUs the spin-diffusion coefficients must be known. The diffusion coefficient of the rigid phase (D_B) can be calculated from the following equation that is valid for the Gaussian line shape [3]:

$$D_B = \frac{1}{12}\sqrt{\frac{\pi}{2\ln 2}} < r^2 > \Delta\nu_{1/2}^B \tag{2}$$

Where $< r^2 >$ is the mean-square distance between the nearest spins (we use a value 0.05 nm^2) [4], $\Delta\nu_{1/2}^B$ is the full width at half height for proton signals of rigid phase obtained by solid-state ^{1}H NMR experiment with a double-quantum (DQ) filter. The spin-diffusion coefficients for mobile phase D_A were calculated using T_2 measurements of mobile fraction (from dipolar filtered Hahn-echo experiment) by method proposed by Mellinger et al [2]. The characteristic size (L) of the polymer matrix, namely the distance between the centers of two closest neighboring dispersed HS domains, i.e. long period, is calculated using equation:

$$L = \frac{d_{dis}}{\sqrt[\varepsilon]{1-\varphi_B}} \tag{3}$$

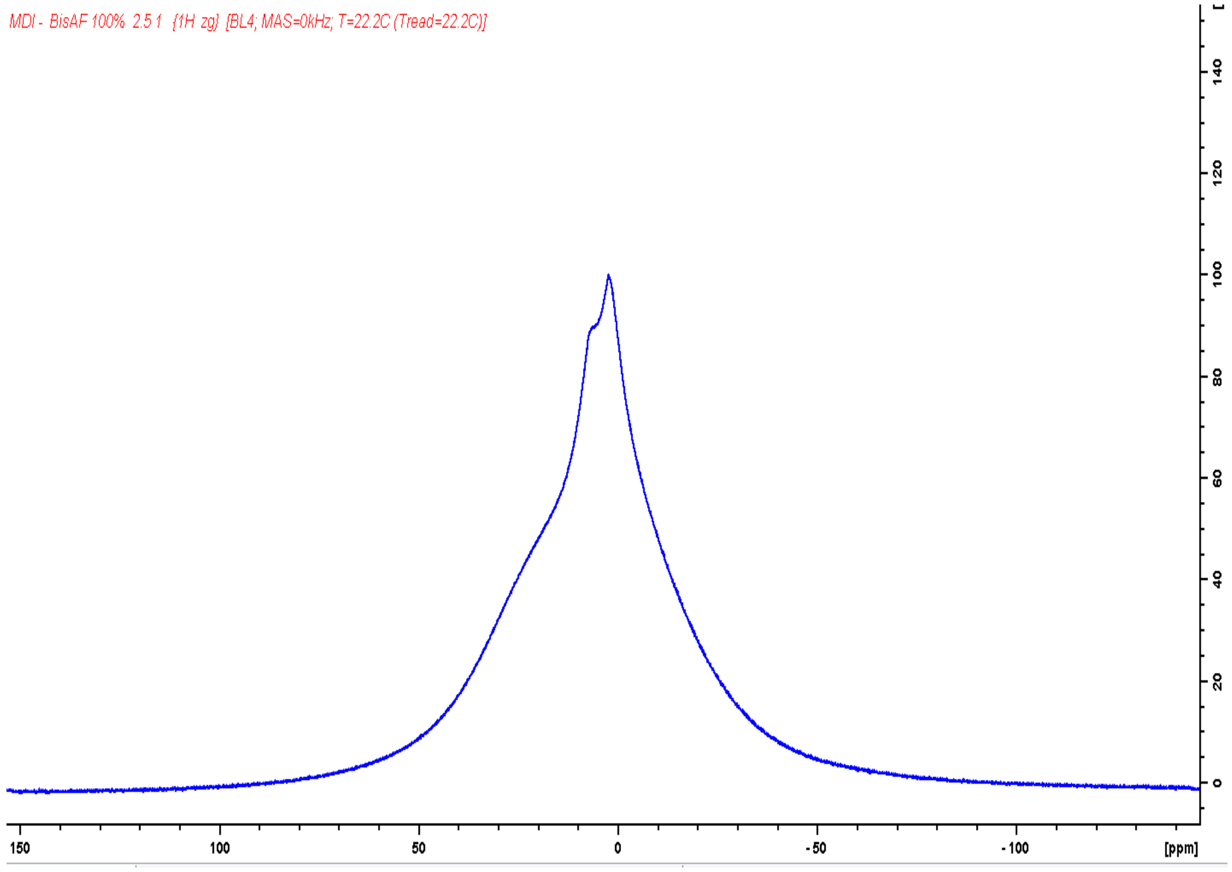

Figure S5. ^{1}H static solid-state NMR spectrum of sample 7.

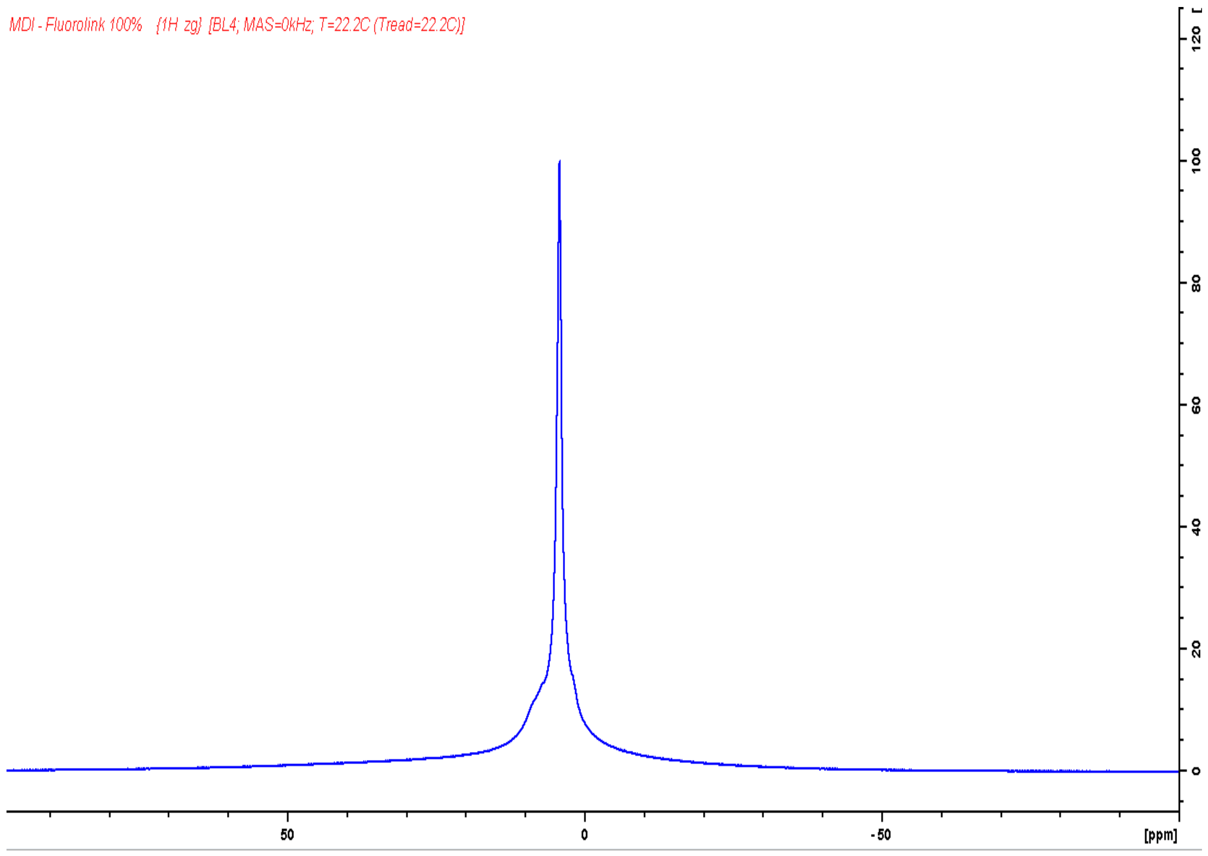

Figure S6. ^{1}H static solid-state NMR spectrum of sample 9.

References

(1) J. Clauss, K. Schmidt-Rohr, H.W. Spiess, Determination of domain sizes in heterogeneous polymers by Solid-State NMR, Acta Polymerica, 44 (1993) 1-17.

(2) F. Mellinger, M. Wilhelm, H.W. Spiess, Calibration of ^{1}H NMR spin diffusion coefficients for mobile polymers through transverse relaxation measurements, Macromolecules, 32 (1999) 4686-4691.

(3) A. Voda, K. Beck, T. Schauber, M. Adler, T. Dabisch, M. Bescher, M. Viol, D. Demco, B. Blumich, Investigation of soft segments of thermoplastic polyurethane by NMR, differential scanning calorimetry and rebound resilience, Polymer Testing, 25 (2006) 203–213.

(4) X.J. Li, W.G. Fu, Y.N. Wang, T.H. Chen, X.H. Liu, H. Lin, P. Sun, Q. Jin, D. Ding, Solid-State NMR characterization of unsaturated polyester thermoset blends containing PEO-PPO-PEO block copolymers, Polymer, 49 (2008) 2886-2897.

Calculation of λ_{DFS}

From these spectra λ_{DFS} is generally evaluated by the following relation:

$$\lambda_{DFS} = \frac{A_b}{A_f + A_b} \qquad (1)$$

where A_b and A_f are the integrated absorbances (ε_b and ε_f are extinction coefficients, assumed to be equal, although the value of ε_b / ε_f is probably between 1.0 and 1.2) of hydrogen bonded and free molecular group of urethane or urea bond, respectively. The C=O bands were deconvoluted using Gaussian band shapes using software adapted Bruker AG for Vertex instrument.

DSC Data

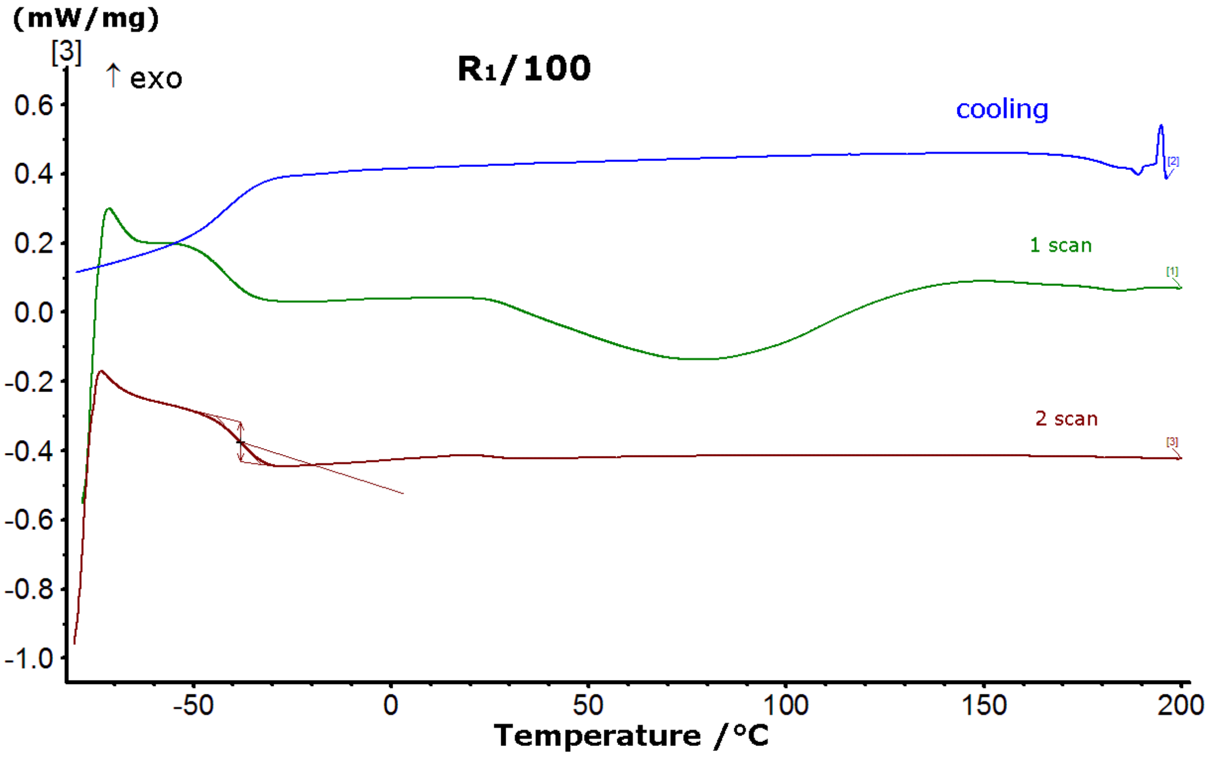

Figure S7. DSC curves for copolymer R1/100.

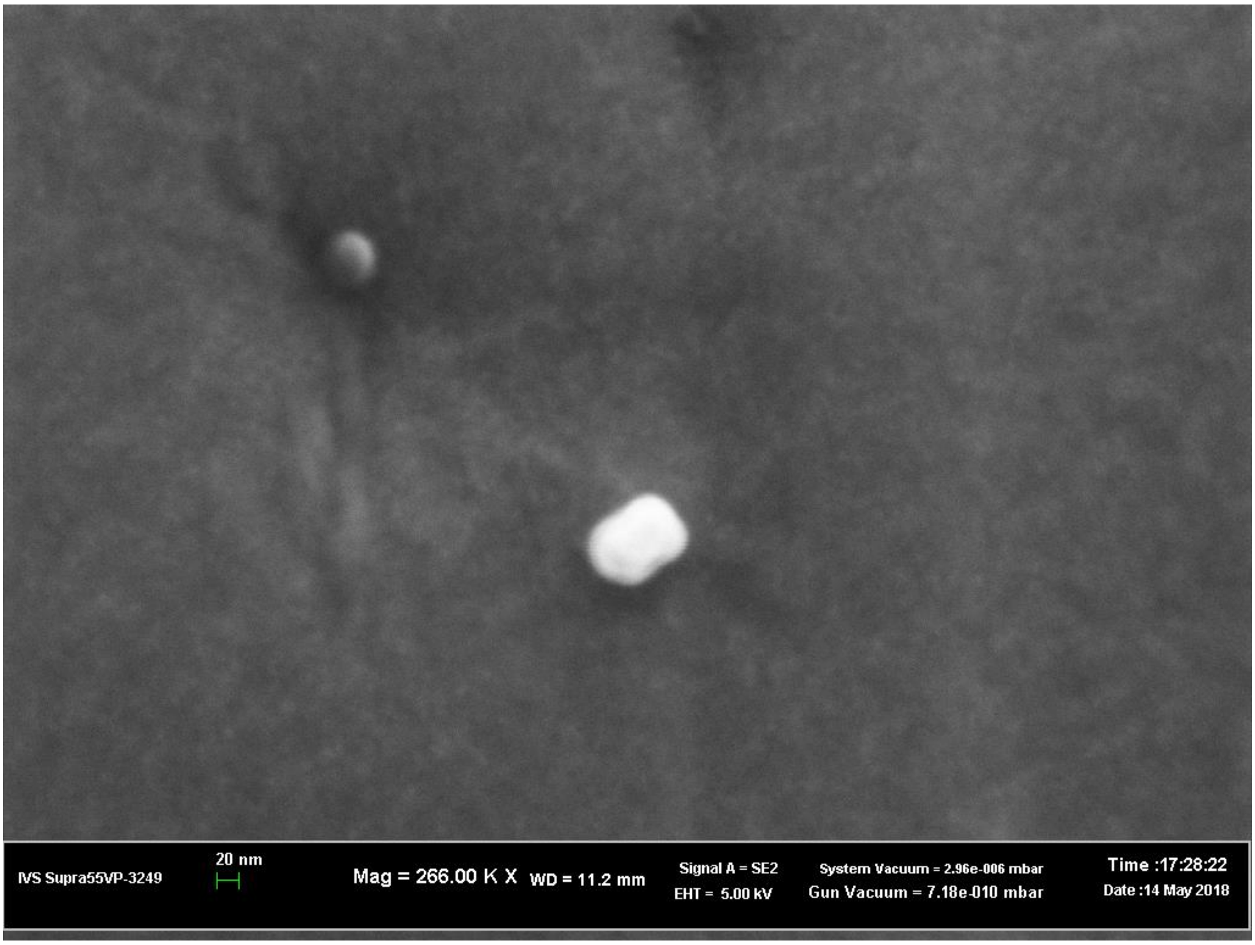
IVS Supra55VP-3249
20 nm
Mag = 266.00 K X
WD = 11.2 mm
Signal A = SE2
EHT = 5.00 kV
System Vacuum = 2.96e-006 mbar
Gun Vacuum = 7.18e-010 mbar
Time :17:28:22
Date :14 May 2018

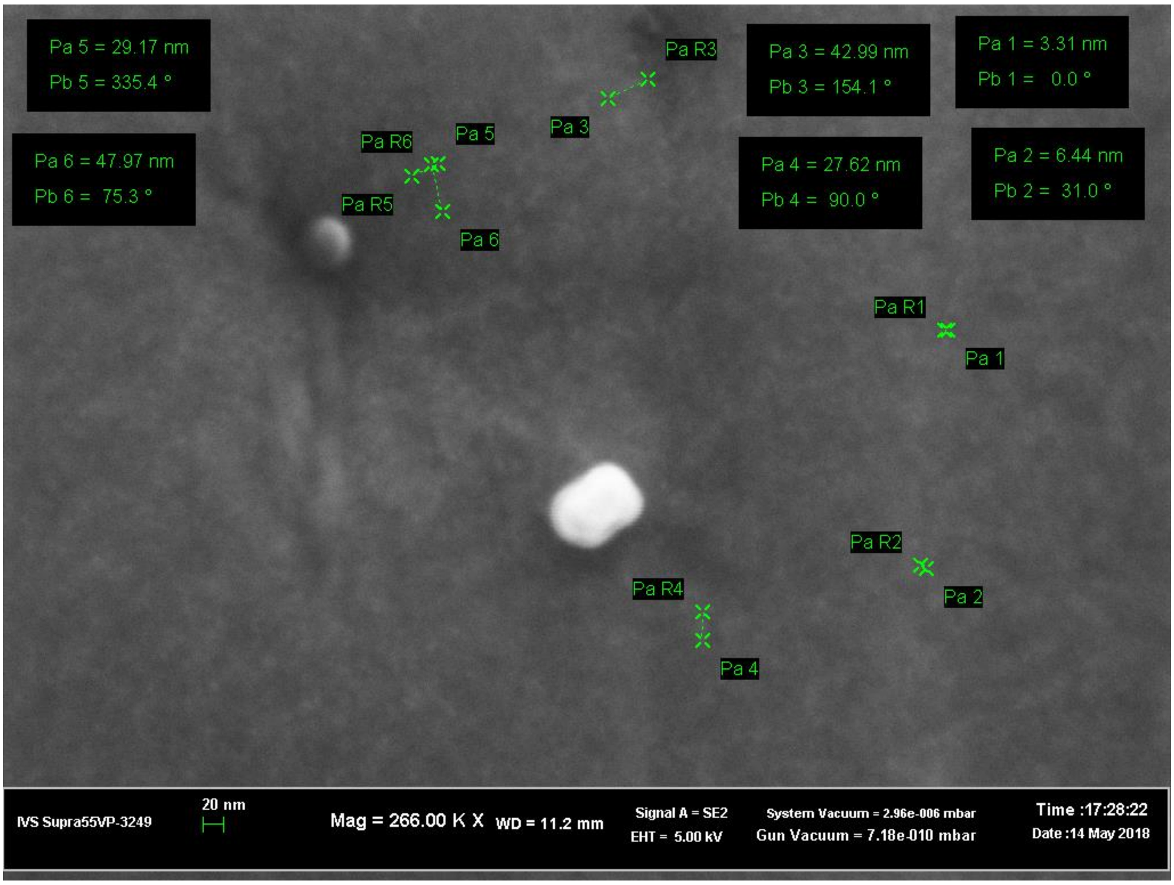
Pa 5 = 29.17 nm
Pb 5 = 335.4 °
Pa 6 = 47.97 nm
Pb 6 = 75.3 °
Pa 3 = 42.99 nm
Pb 3 = 154.1 °
Pa 1 = 3.31 nm
Pb 1 = 0.0 °
Pa 4 = 27.62 nm
Pb 4 = 90.0 °
Pa 2 = 6.44 nm
Pb 2 = 31.0 °
Pa R3
Pa 3
Pa R6
Pa 5
Pa R5
Pa 6
Pa R1
Pa 1
Pa R2
Pa 2
Pa R4
Pa 4
IVS Supra55VP-3249
20 nm
Mag = 266.00 K X
WD = 11.2 mm
Signal A = SE2
EHT = 5.00 kV
System Vacuum = 2.96e-006 mbar
Gun Vacuum = 7.18e-010 mbar
Time :17:28:22
Date :14 May 2018

Figure S8. SEM images of sample R1/100. The measurement of length and diameter of cylindrical domains.

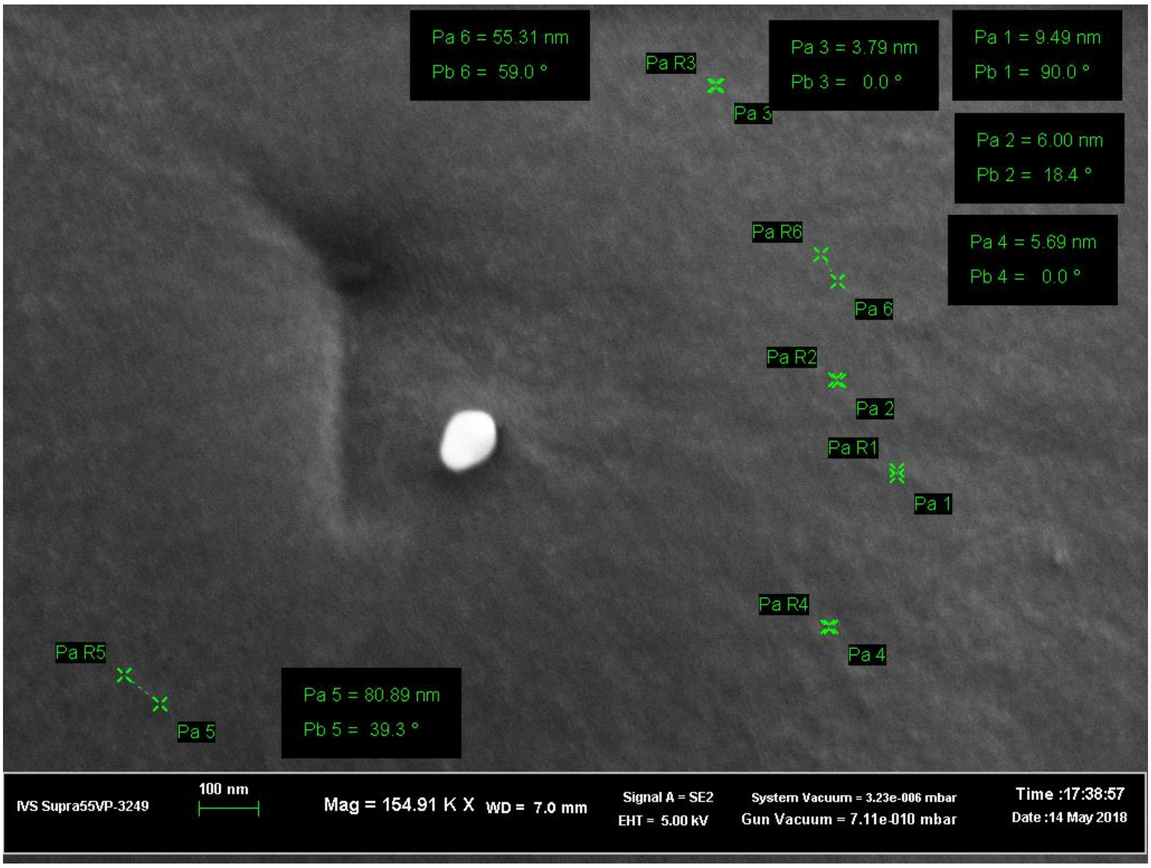

Figure S9. SEM image of sample R1/50R3/50

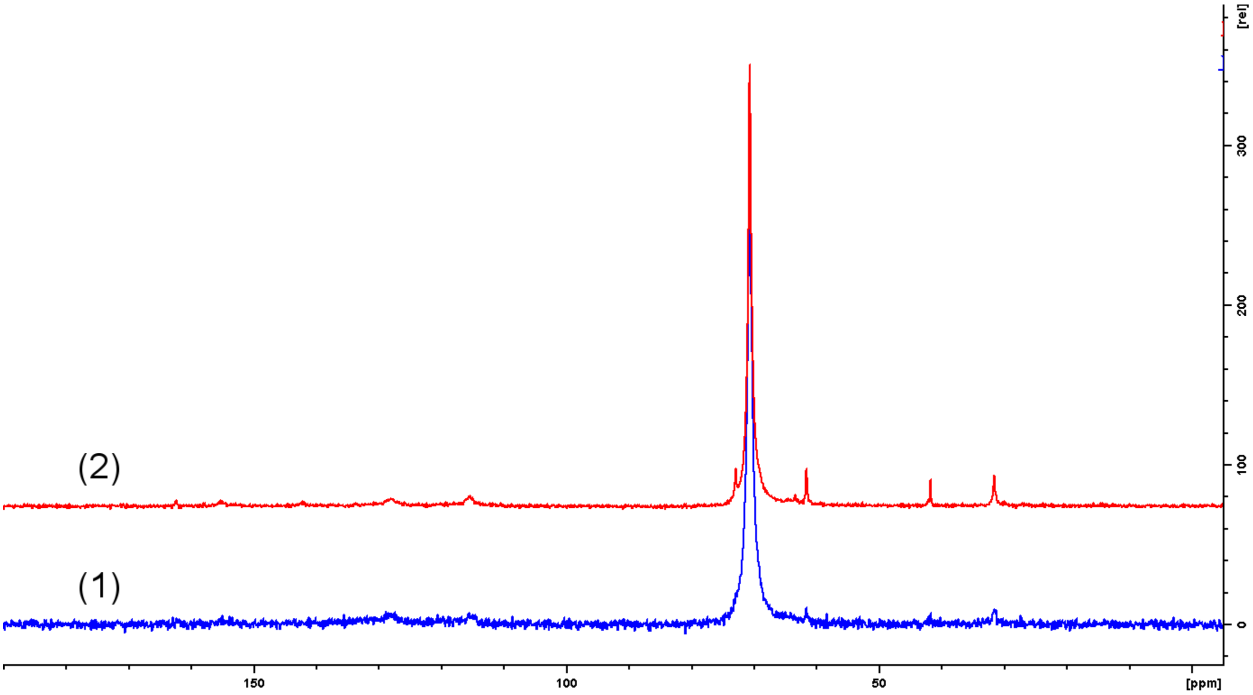

Figure S10. ^{13}C CP/MAS (1) and ^{13}C CPSP/MAS (2) spectra of sample R_1/70/R_3/30

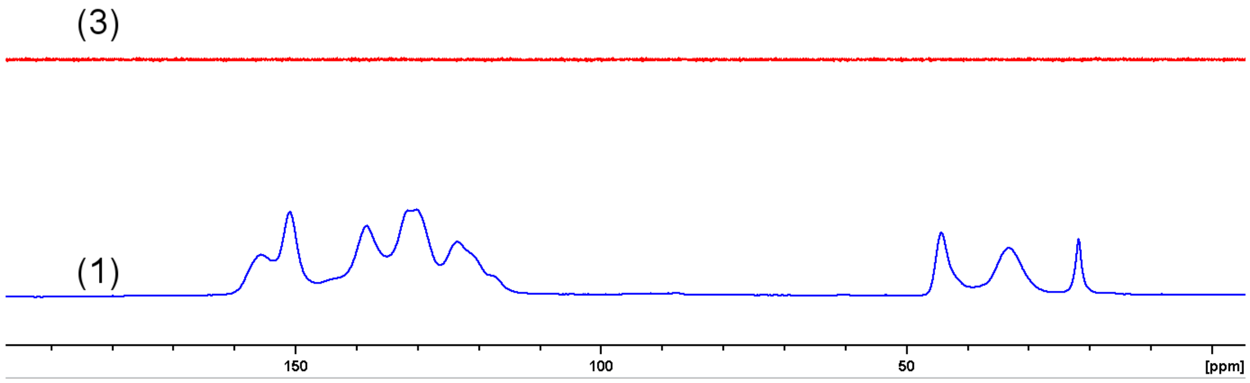

Figure S11. ^{13}C CP/MAS (1) and ^{13}C direct-polarization MAS (3) spectra of sample $R_3/100$

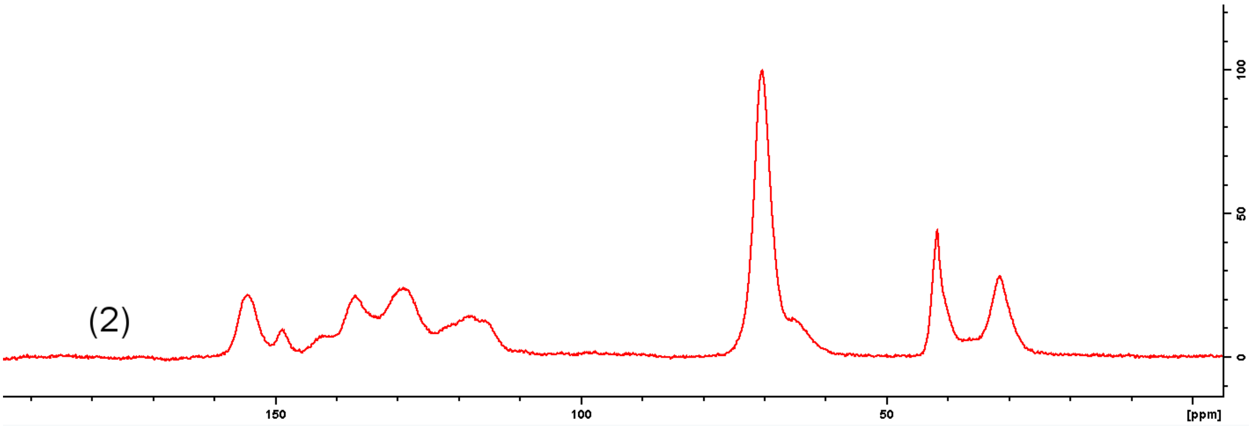

Figure S12. ^{13}C CPSP/MAS (2) spectrum of sample $R_1/30/R_3/70$

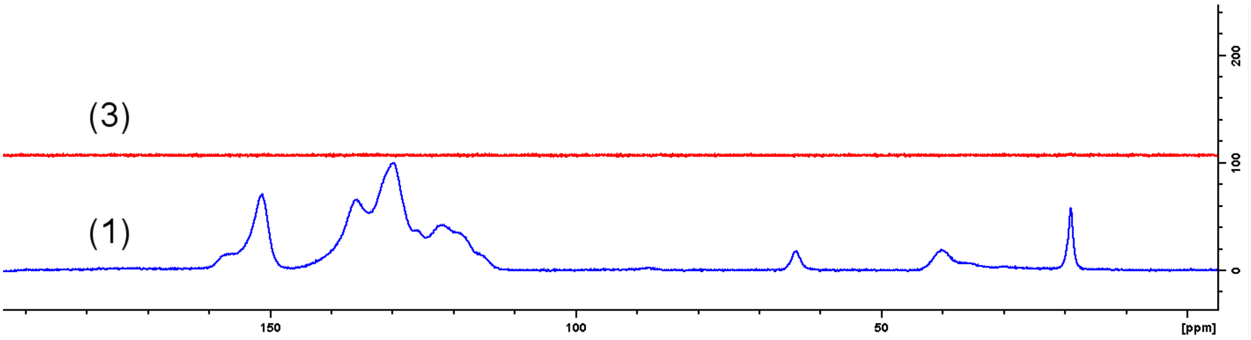

Figure S13. ^{13}C CP/MAS (1) and ^{13}C direct-polarization MAS (3) spectra of sample $R_4/100$

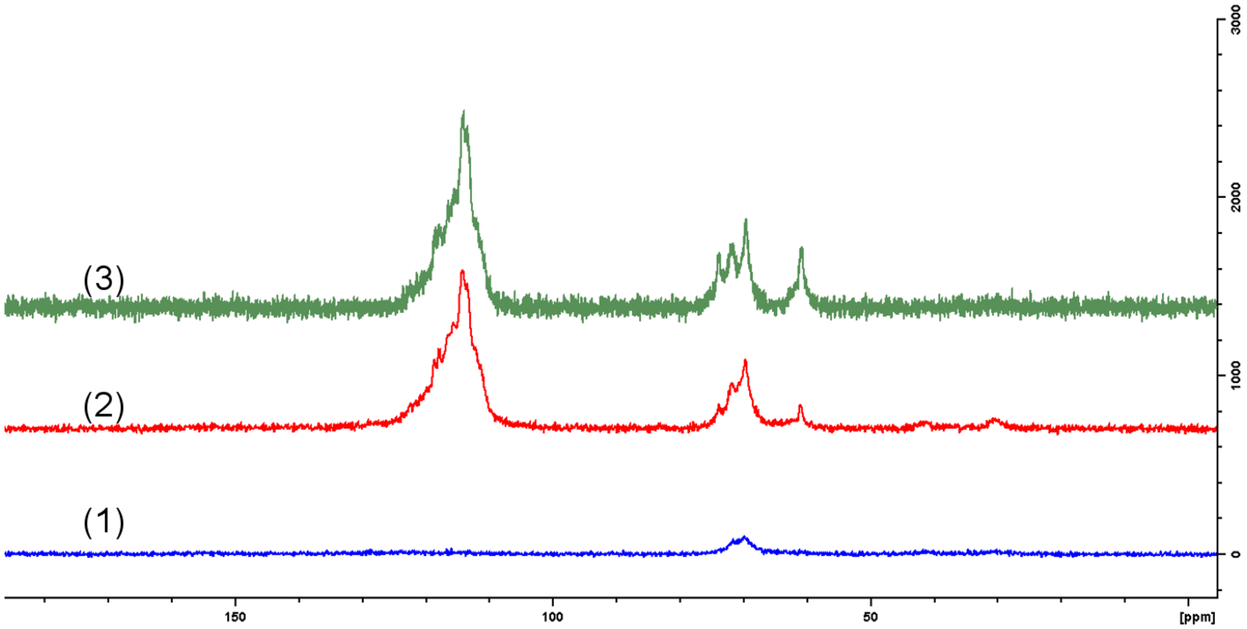

Figure S14. ^{13}C CP/MAS (1); ^{13}C CPSP/MAS (2) and ^{13}C direct-polarization MAS (3) spectra of sample $R_2/50/R_3/50$

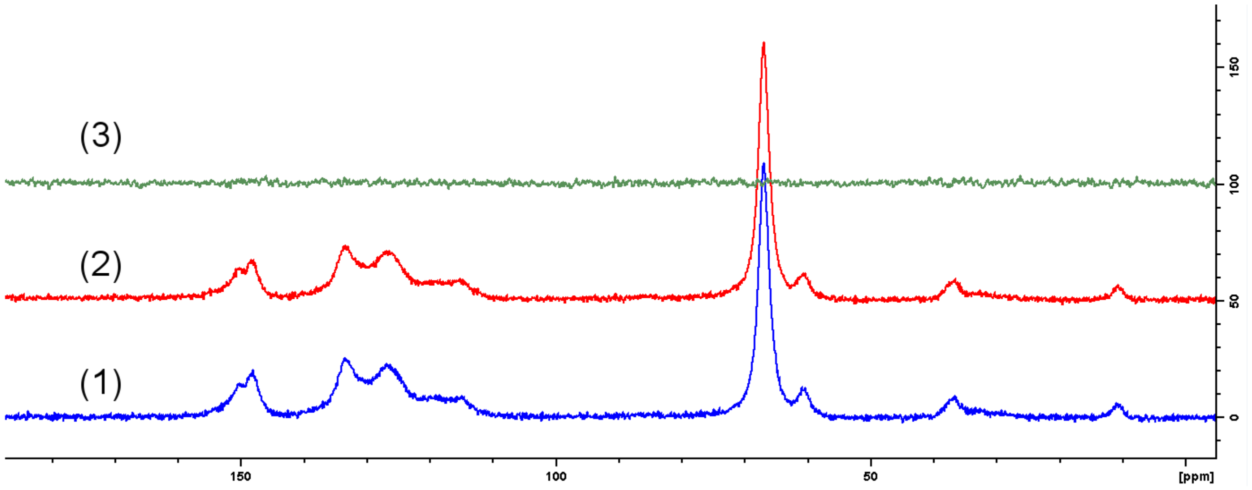

Figure S15. ^{13}C CP/MAS (1); ^{13}C CPSP/MAS (2) and ^{13}C direct-polarization MAS (3) spectra of sample $R_1/50/R_4/50$

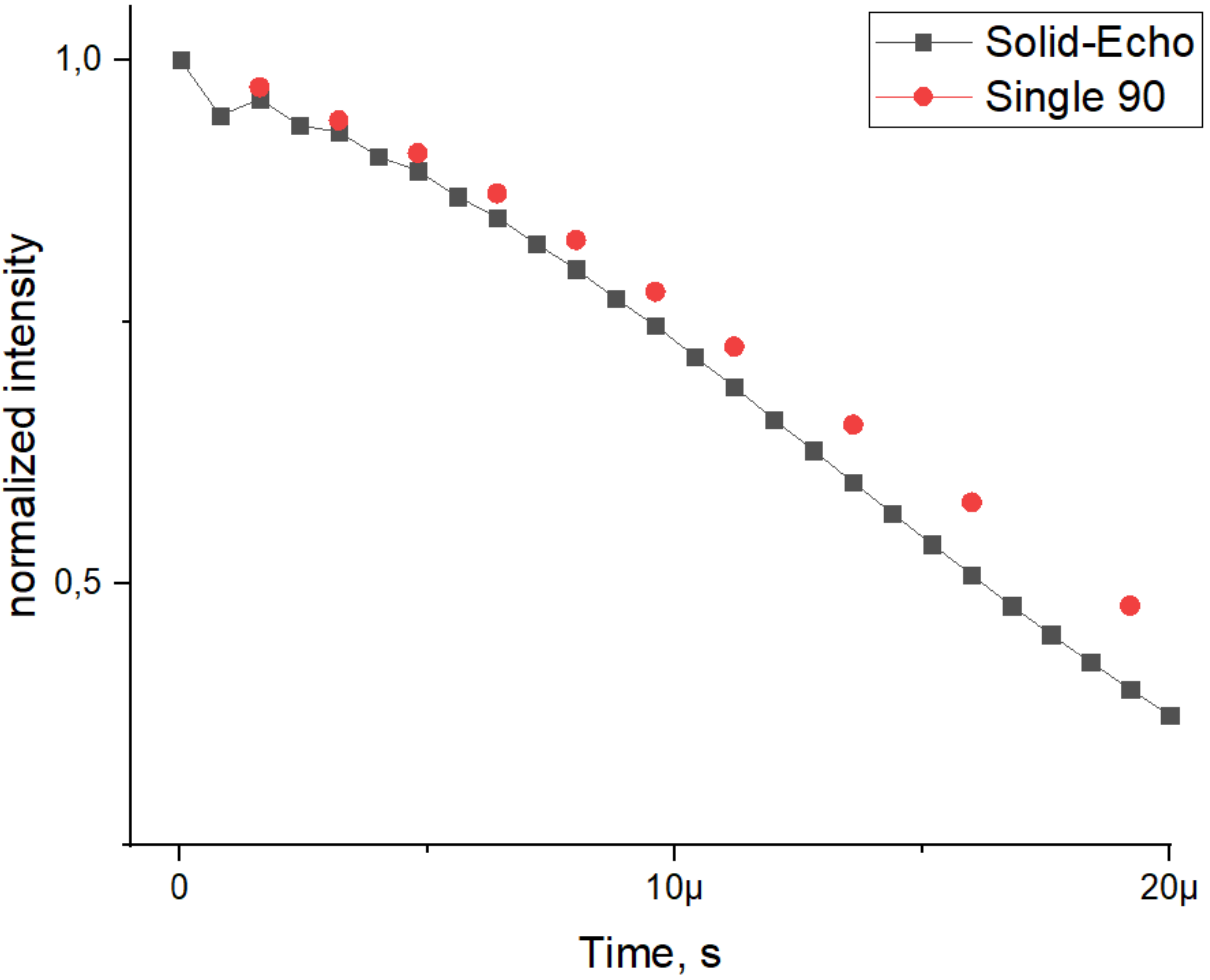

Figure S16. Solid echo (black) and single pulse (red) ^{1}H signal for sample R3/100, with background signal subtracted. The single-pulse signal was horizontally shifted by 5 □s to account for the dead time and vertically scaled by a factor of 0.98. Good agreements for both the fast-decaying and the slow-decaying components are observed, as the black and red curves almost completely overlap.
